# Supplementary material for: Astrocytes detect and upregulate transmission at inhibitory synapses of somatostatin interneurons onto pyramidal cells
Source: Nat Commun. 2018 Oct 12;9:4254. doi: 10.1038/s41467-018-06731-y (PMC6185912; doi:10.1038/s41467-018-06731-y)
Supplement: Supplementary file 1 — Supplementary Information [file 41467_2018_6731_MOESM1_ESM.pdf]

Supplementary information

Astrocytes detect and upregulate transmission at inhibitory synapses of somatostatin interneurons onto pyramidal cells

Matos et al.

Matos et al. Supplementary Figure 1

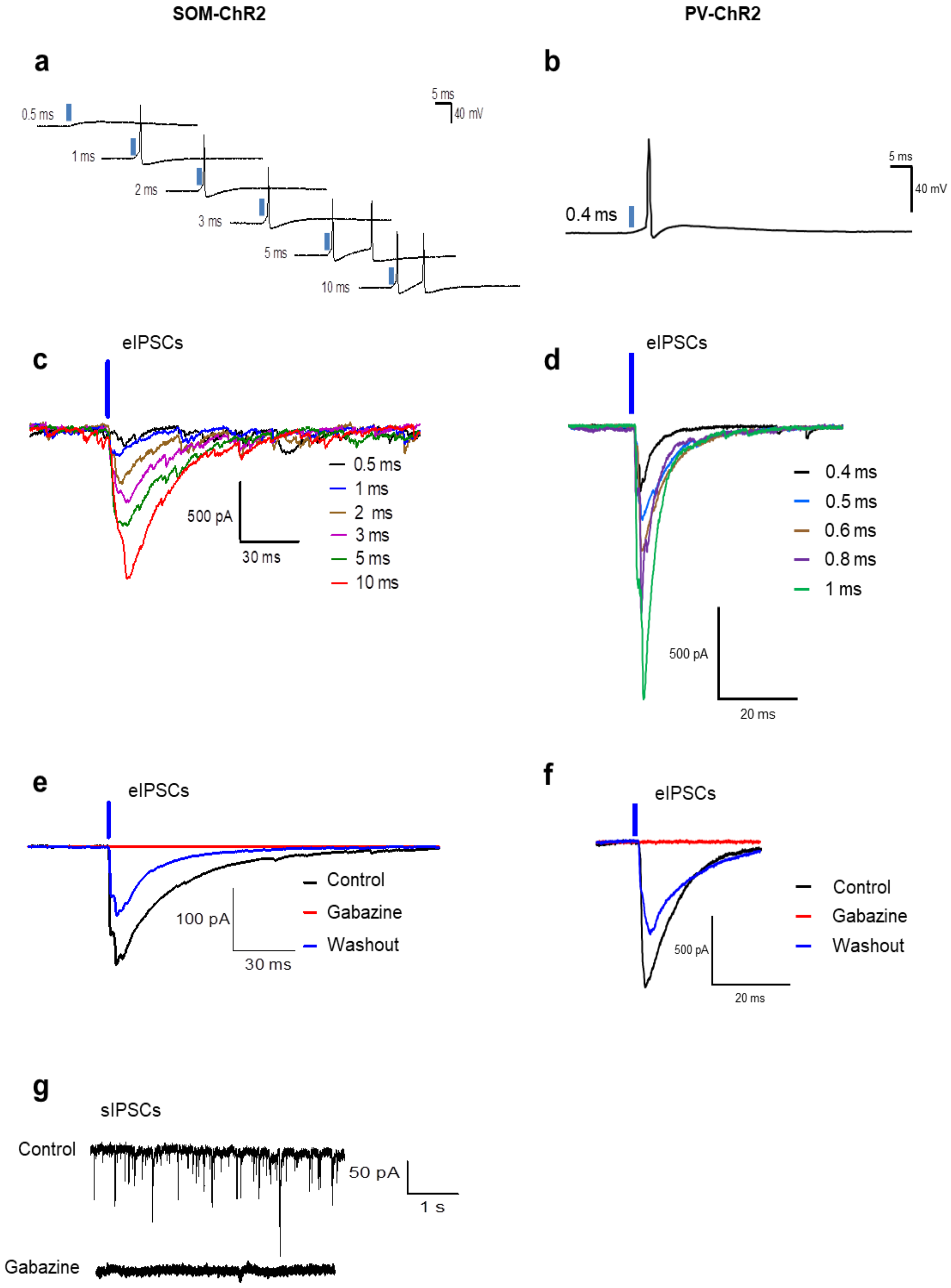

Supplementary Figure 1. Activation of SOM-INs or PV-INs, and inhibition of pyramidal cells, by optogenetic stimulation in SOM-ChR2/EYFP or PV-ChR2/EYFP mice respectively. a Representative depolarizing responses and action potential firing evoked in a SOM-IN by optogenetic stimulation with blue light (pulse duration 0.5-10 ms; 0.1 Hz) during whole-cell current-clamp recordings of YFP-expressing SOM-INs (n = 4) in slices from SOM-ChR2/EYFP mice. **b** Representative trace with depolarization and action potential firing evoked in a PV-IN by optogenetic stimulation with blue light (0.4ms pulse; 0.1 Hz) during whole-cell current-clamp recording of YFP-expressing PV-INs in slice from PV-ChR2/EYFP mice. **c** SOM-IPSCs from a representative pyramidal cell evoked by the same optogenetic stimulation (0.5-10 ms pulse; 0.1 Hz) of SOM-INs. **d** PV-IPSCs recorded in a representative pyramidal cell evoked by optogenetic stimulation (0.4–1 ms pulse; 0.1 Hz) of PV-INs. **e-f** Traces from representative pyramidal cells showing the reversible block of SOM-IPSCs **e** and PV-IPSCs **f** by the $GABA_A$R antagonist Gabazine (5 μM, red trace), confirming the $GABA_A$R-mediated inhibition by SOM-INs and PV-INs. **g** Traces from a representative CA1 pyramidal cell showing the block of spontaneous IPSCs (sIPSCs) after 5 min perfusion with Gabazine.

Matos et al. Supplementary Figure 2

a **b**

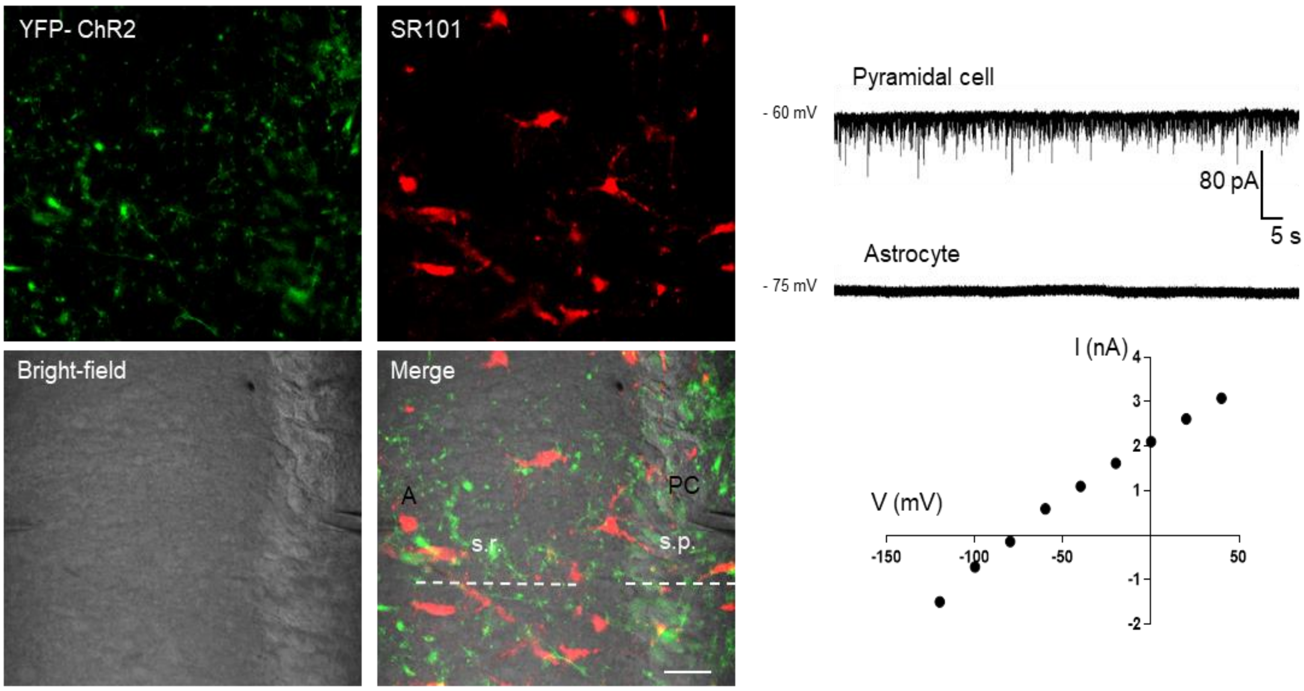

c

	SOM-eIPSC amplitude (pA ± SEM)	sIPSC amplitude (pA ± SEM)	sIPSC frequency (Hz ± SEM)	sIPSC rise tau (ms ± SEM)	sIPSC decay tau (ms ± SEM)	R_m (MΩ ± SEM)	V_m (mV ± SEM)
**Control**	-	57.47 ± 10.04	11.00 ± 1.04	4.00 ± 2.00	15.02 ± 4.20	192.33 ± 13.30	61.04 ± 2.01
**SR101**	95.10 ± 17.24	52.03 ± 6.07	13.02 ± 3.40	3.48 ± 1.00	14.44 ± 6.20	196.83 ± 44.30	60.43 ± 1.03
**P value**	> 0.05	> 0.05	> 0.05	> 0.05	> 0.05	> 0.05	> 0.05

Supplementary Figure 2. Identification of astrocytes with SR101 and lack of effect on pyramidal cell spontaneous IPSCs and membrane properties. a Representative images of CA1 *stratum radiatum* and *pyramidale*, depicting astrocytes labelled by SR101 (250 nM; red), processes of YFP-positive SOM-INs (green) and cells in bright-field. Images show two simultaneous whole-cell recordings obtained from an astrocyte (A in merged image) and a pyramidal cell (PC in merged image). Scale bar 10 µm. **b** Representative dual voltage-clamp recordings of a pyramidal cell and an SR101-labelled astrocyte, illustrating the numerous fast $GABA_AR$-dependent spontaneous IPSCs in the pyramidal cell (upper trace) and the absence of synaptic currents in the astrocyte (lower trace). Below, I-V plot from a hippocampal astrocyte, indicating a linear current-voltage relationship in response to current injections. **c** Summary table of pyramidal cell IPSCs properties, as well as membrane resistance (R_m) and membrane potential (V_m), showing no effect of application of SR101 dye (n = 10) (unpaired

two-sample Student T-test). A: astrocyte; PC: pyramidal cell; s.p.: *stratum pyramidale*; s.r.: *stratum radiatum*.

Matos et alSupplementary Figure 3

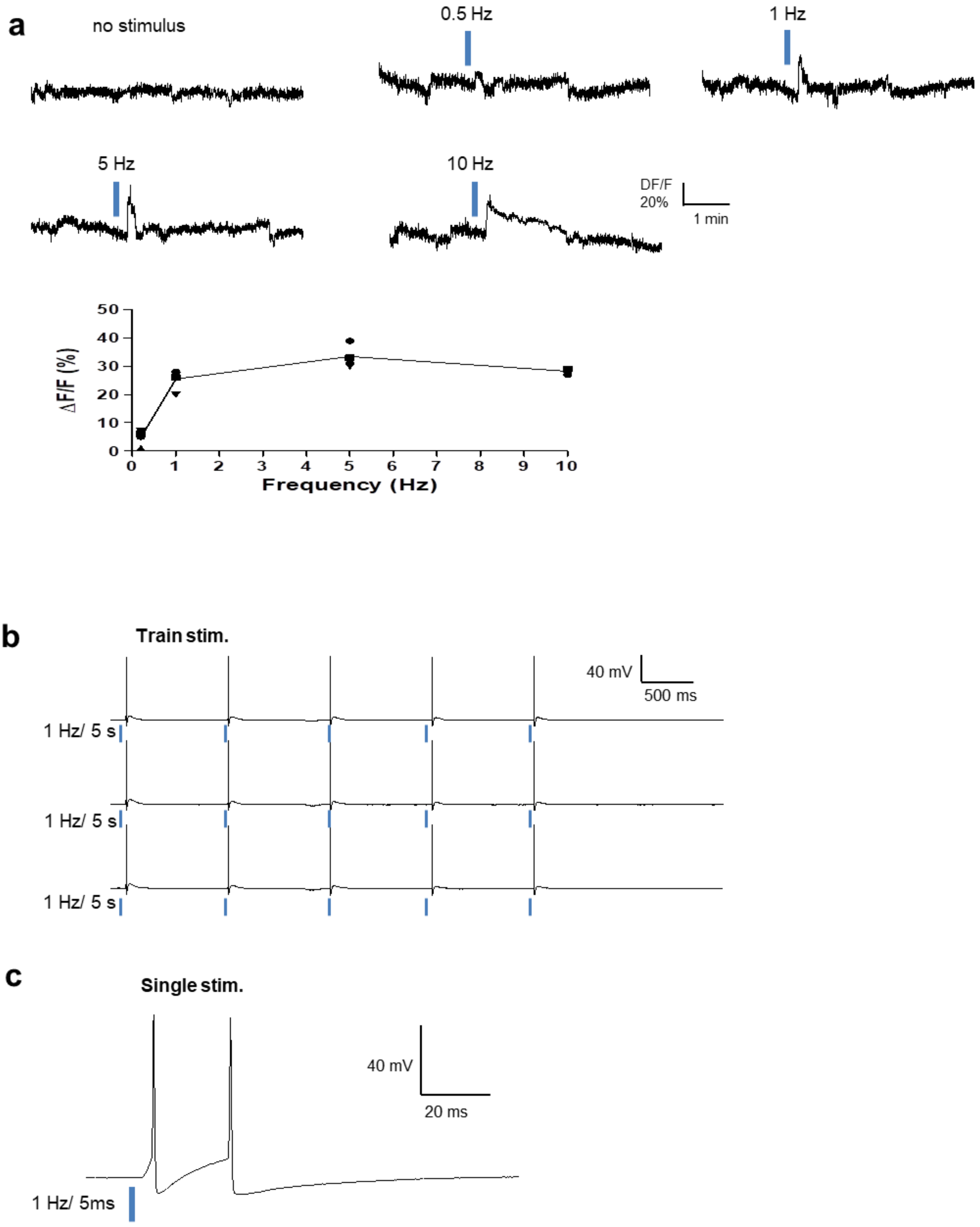

Supplementary Figure 3. Optogenetic stimulation protocols and action potential firing in SOM-INs to evoke Ca^{2+} signals in astrocytes. **a** Top: Representative Ca^{2+} responses from an astrocyte process evoked by different optogenetic stimulation protocols (5ms pulses at 0-10 Hz for 5 s), showing that 1 Hz protocol was sufficient to elicit large Ca^{2+} responses. Bottom: Graph depicting the Ca^{2+} transients amplitude as a function of optogenetic stimulation frequency (n = 3). **b** Representative whole-cell current-clamp recordings of SOM-INs, showing the action potential firing evoked in SOM-INs by the optogenetic train stimulation (5ms pulses at 1Hz for 5 sec) used for inducing Ca^{2+} signals in astrocytes (n = 4). **c** Representative whole-cell current-clamp recording of SOM-INs, showing the depolarizing response evoked in SOM-INs by optogenetic single stimulation (single 5ms pulse at 1Hz).

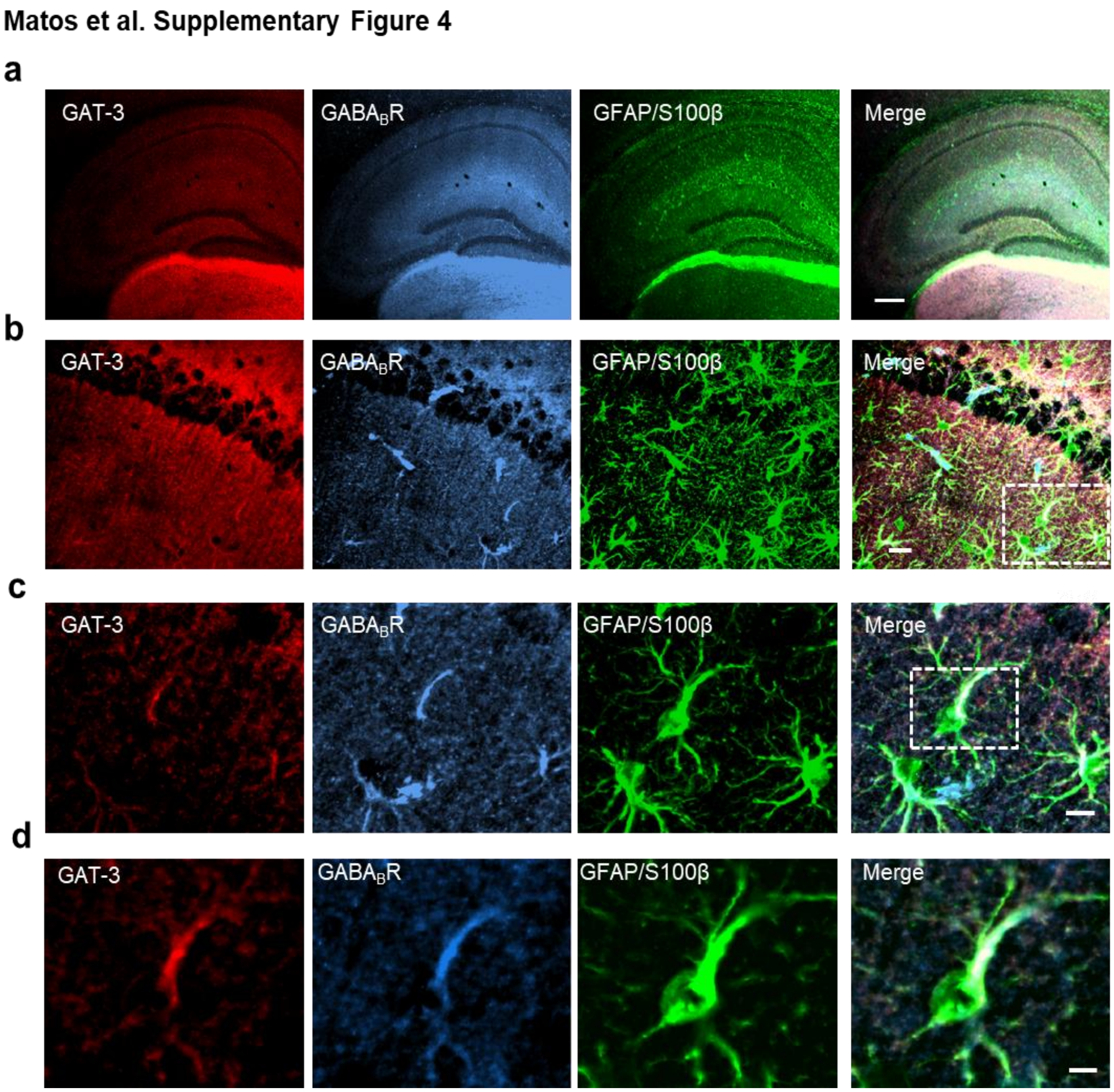

Supplementary Figure 4. GAT-3 and $GABA_BR$ co-localize in astrocytic processes. a Low-magnification immunofluorescence images, depicting hippocampal immunolabelling for GAT-3 (red), $GABA_BR$ (blue) and astrocyte-specific GFAP/S100β (green), with the merged images at right. Scale bar 100 µm **b-c** Representative z-stack images at higher magnification from *stratum pyramidale* and *radiatum* regions, showing $GABA_BR$ (blue) and GAT-3 (red) staining co-localized in the GFAP/S100β-immunoreactive astrocytic processes (green). Boxed region in **b** corresponds to high-magnification images in **c**. Scale bars 20 µm (**b**), 10 µm (**c**). **d** Different example of high-magnification image in *stratum radiatum* showing GFAP/S100β-immunoreactive astrocytic processes (green) with co-localized $GABA_BR$ (blue) and GAT-3 (red) staining**.** Scale bar 5 µm.

Figure	Panel	Sample size	p value	Test
**1**	**h**	Vehicle n = 8<br>DPCPX n = 8<br>AMP-CP n = 7	 p < 0,01<br>p < 0,01 *vs*, control ; p > 0,05 *vs*, DPCPX	One-way ANOVA Tukey's post hoc test
	**i**	AMP-CP n = 7<br>DPCPX + AMP-CP n = 6	p < 0,01<br>p < 0,01	Kruskal-Wallis Dunn's post hoc test
**2**	**e**	BAPTA 0,1 mM n = 8<br>BAPTA 20 mM n = 12	 p < 0,001	Kruskal-Wallis Dunn's post hoc test
**3**	**k**	Vehicle n = 7<br>SNAP-5114 n = 8<br>CGP55845 n = 7<br>CGP55845 + SNAP-5114 n = 7	p > 0,05<br>p < 0,001<br>p < 0,01 *vs*, control ; p < 0,05 *vs*, SNAP-5114<br>p < 0,001 *vs*, control ; p < 0,01 *vs*, CGP55845	One-way ANOVA Tukey's post hoc test
**4**	**d**	Vehicle n = 7<br>SNAP-5114 n = 6<br>CGP55845 n = 7	p > 0,05<br>p < 0,01<br>p > 0,05	Kruskal-Wallis Dunn's post hoc test
	**h**	BAPTA n = 8<br>BAPTA + (S)-SNAP-5114 n = 8	p < 0,001<br>p < 0,001 ; p > 0,05 *vs*, BAPTA	One-way ANOVA Dunn's post hoc test
**5**	**c**	(S)-SNAP-5114 n = 6<br>(S)-SNAP-5114 + DPCPX n = 6	p < 0,001<br>p < 0,001 ; p > 0,05 *vs*, (S)-SNAP-5114	One-way ANOVA Tukey's post hoc test
	**d**	(S)-SNAP-5114 n = 4<br>(S)-SNAP-5114 + N^6CPA n = 4<br>(S)-SNAP-5114 + N^6CPA + DPCPX n = 4	p > 0,05 *vs*, (S)-SNAP-5114 + N^6CPA + DPCPX<br>p < 0,001 *vs*, (S)-SNAP-5114 ; p < 0,001 *vs*, (S)-SNAP-5114 + N^6CPA + DPCPX<br>p > 0,05 *vs*, (S)-SNAP-5114 + N^6CPA	Friedman test Dunn's post hoc test
	**f**	Vehicle n = 5<br>N^6CPA n = 5	p > 0,05<br>p > 0,05	Wilcoxon signed-rank test
**6**	**f**	Vehicle n = 7<br>DPCPX n = 7<br>SNAP-5114 n = 6	p > 0,05<br>p > 0,05<br>p > 0,05	Friedman test Dunn's post hoc test
**7**	**c**	DPCPX n = 7	sIPSC Amp p < 0,05 *vs*, control	One-way ANOVA Tukey's post hoc test
	**f**	AMP-CP n = 7 (each)	p > 0,05	One-way ANOVA Tukey's post hoc test
	**i**	(S)-SNAP-5114 n = 7	sIPSC Amp p < 0,01 *vs*, control<br>sIPSC Freq p < 0,05 *vs*, control	One-way ANOVA Tukey's post hoc test
	**l**	20 min post-BAPTA n = 6<br>(S)-SNAP-5114 n = 6	sIPSC Amp p < 0,001 *vs*, control<br>sIPSC Freq p < 0,05 *vs*, control<br>sIPSC Amp p < 0,001 *vs*, control<br>sIPSC Freq p < 0,05 *vs*, control	One-way ANOVA Tukey's post hoc test

Supplementary Table 1. Statistical analyses.
